# Aortic Fibrosis in Insulin-Sensitive Mice with Endothelial Cell-Specific Deletion of *Ceacam1* Gene

**DOI:** 10.3390/ijms23084335

**Published:** 2022-04-14

**Authors:** Raghd Abu Helal, Harrison T. Muturi, Abraham D. Lee, Wei Li, Hilda E. Ghadieh, Sonia M. Najjar

**Affiliations:** 1 Department of Biomedical Sciences, Heritage College of Osteopathic Medicine, Ohio University, Athens, OH 45701, USA; ra095216@ohio.edu (R.A.H.); muturi@ohio.edu (H.T.M.); hg36@aub.edu.lb (H.E.G.); 2 Center for Diabetes and Endocrine Research, College of Medicine, University of Toledo, Toledo, OH 43606, USA; abraham.lee2@utoledo.edu; 3 School of Exercise and Rehabilitation Sciences, College of Health and Human Services, University of Toledo, Toledo, OH 43606, USA; 4 Department of Biomedical Sciences, Joan C. Edwards School of Medicine at Marshall University, Huntington, WV 25755, USA; liwe@marshall.edu; 5 Department of Biomedical Sciences, Faculty of Medicine and Medical Sciences, University of Balamand, El-Koura P.O. Box 100, Lebanon; 6 Diabetes Institute, Heritage College of Osteopathic Medicine, Ohio University, Athens, OH 45701, USA

**Keywords:** insulin resistance, inflammation, oxidative stress, atheroma, fibrosis, fatty streaks

## Abstract

(1) Background: Mice with global *Ceacam1* deletion developed plaque-like aortic lesions even on C57BL/6J background in the presence of increased endothelial cell permeability and insulin resistance. Loss of endothelial *Ceacam1* gene caused endothelial dysfunction and reduced vascular integrity without affecting systemic insulin sensitivity. Because endothelial cell injury precedes atherosclerosis, we herein investigated whether the loss of endothelial *Ceacam1* initiates atheroma formation in the absence of insulin resistance. (2) Methods: Endothelial cell-specific *Ceacam1* null mice on *C57BL/6J.Ldlr^−/−^* background (*Ldlr^−/−^VECadCre+Cc1^fl/fl^*) were fed an atherogenic diet for 3–5 months before metabolic, histopathological, and en-face analysis of aortae were compared to their control littermates. Sirius Red stain was also performed on liver sections to analyze hepatic fibrosis. (3) Results: These mice displayed insulin sensitivity without significant fat deposition on aortic walls despite hypercholesterolemia. They also displayed increased inflammation and fibrosis. Deleting *Ceacam1* in endothelial cells caused hyperactivation of VEGFR2/Shc/NF-κB pathway with resultant transcriptional induction of NF-κB targets. These include IL-6 that activates STAT3 inflammatory pathways, in addition to endothelin-1 and PDGF-B profibrogenic effectors. It also induced the association between SHP2 phosphatase and VEGFR2, downregulating the Akt/eNOS pathway and reducing nitric oxide production, a characteristic feature of endothelial dysfunction. Similarly, hepatic inflammation and fibrosis developed in *Ldlr^−/−^VECadCre+Cc1^fl/fl^* mice without an increase in hepatic steatosis. (4) Conclusions: Deleting endothelial cell *Ceacam1* caused hepatic and aortic inflammation and fibrosis with increased endothelial dysfunction and oxidative stress in the presence of hypercholesterolemia. However, this did not progress into frank atheroma formation. Because these mice remained insulin sensitive, the study provides an in vivo demonstration that insulin resistance plays a critical role in the pathogenesis of frank atherosclerosis.

## 1. Introduction

Atherosclerotic cardiovascular disease remains a major cause of death worldwide [1]. Several risk factors predispose to atherosclerosis, most importantly the metabolic syndrome: a constellation of cardiometabolic abnormalities that include visceral obesity, hypertension, and non-alcoholic fatty liver disease (NAFLD) [2]. Common molecular mechanisms include insulin resistance and dyslipidemia characterized by elevated serum triglycerides, decreased HDL cholesterol, and a remarkable increase in small dense LDL cholesterol [3,4]. Whether insulin resistance leads to atherosclerosis independently of accompanying dyslipidemia remains unclear, owing in part to the lack of an experimental model. Most commonly used atherosclerosis-prone mouse models are the apolipoprotein E-deficient (*ApoE^−/−^*) and LDL receptor-deficient (*Ldlr^−/−^*) mice, which exhibit a phenotype driven by extreme hypercholesterolemia that exceeds the level found in most patients with atherosclerosis and can act as the sole atherogenic factor that masks the potential effect of insulin resistance in the pathogenesis of atherosclerosis [5].

Insulin resistance is an independent risk factor for atherosclerosis [3,6,7]. It exerts an atherogenic effect on the vascular endothelium in association with endothelial dysfunction [4,8,9] that is manifested by compromised vasodilation and increased leukocyte-endothelium adhesiveness [8,10], both of which are caused by reduced nitric oxide (NO) bioavailability [4]. Insulin resistance also increases the vasoconstrictor tone by upregulating NF-kB transcriptional activity to induce the expression of endothelin-1 [11] and of pro-inflammatory cell adhesion molecules, such as vascular cell adhesion molecule-1 (VCAM-1) [12]. 

Carcinoembryonic antigen-related cell adhesion molecule 1 (CEACAM1), a transmembrane glycoprotein and a common substrate of insulin receptor and vascular epidermal growth factor receptor 2 (VEGFR2) [13,14], is ubiquitously expressed. In hepatocytes, it regulates insulin action by promoting insulin clearance [15]. Accordingly, its global and liver-specific biallelic deletion cause hepatic insulin resistance and increased hepatic de novo lipogenesis that leads to VLDL-triglyceride redistribution to the white adipose tissue to contribute to visceral obesity, followed by lipolysis and inflammation with resultant systemic insulin resistance [16]. In addition, global *Cc1^−/−^* null mice fed a regular chow diet manifested cardiac and endothelial dysfunctions, and aortic plaque-like lesions with macrophage and collagen deposition and restricted fat accumulation, likely owing to its limited pro-atherogenic hypercholesterolemia [17]. Conversely, liver-specific reconstitution of CEACAM1 restored insulin sensitivity and reversed hepatic steatosis in *Cc1^−/−^* mice [18]. It also reversed their endothelial and cardiovascular dysfunctions in parallel to restoring nitric oxide (NO) bioavailability and reduced endothelial-leukocyte adhesion [19]. This prevented diet-induced non-alcoholic steatohepatitis (NASH) that is often linked to atherosclerosis [20]. In support of the role of hepatic CEACAM1 in atherosclerosis, mice with liver-specific *Ceacam1* deletion developed NASH and atherogenic plaques when propagated on *Ldlr^−/−^* background and fed a high-cholesterol diet [21]. While this mouse model provided an in vivo evidence of how hyperinsulinemia-driven insulin resistance links NASH to atherosclerosis in mice with pro-atherogenic hypercholesterolemia [21], it did not dissect out the role of insulin resistance independently of dyslipidemia in the development of aortic plaques. On the other hand, *ApoE^−/−^* mice with whole-body insulin receptor haploinsufficiency developed hyperinsulinemia in response to impaired insulin clearance [22]. However, in the absence of insulin resistance and significant changes in dyslipidemia, they developed comparable levels of aortic plaques to their *ApoE^−/−^* controls [22]. While this mouse demonstrated that hyperinsulinemia without insulin resistance did not exert a pro-atherogenic effect, it failed to dissociate insulin resistance from dyslipidemia in the pathogenesis of atherosclerosis. 

Endothelial dysfunction can also be caused by endothelial cell injury, which in turn can play an essential role in the pathogenesis of atherosclerosis [23]. Loss of endothelial *Ceacam1* disturbed the endothelial barrier integrity and caused permeability owing to the dissociation of VE–cadherin/β-catenin complexes at cell junctions [24]. Endothelial cell injury in *BL6.VECadCre+Cc1^fl/fl^* mice with endothelial-specific deletion of *Ceacam1* was accompanied by persistent insulin sensitivity. In response to increased paracellular insulin transport, these mice exhibited intact glucose turnover [25]. They also manifested enhanced fat storage in adipocytes despite the development of oxidative stress and increased NF-kB–dependent production of pro-inflammatory cytokines (TNFα) and pro-fibrogenic effectors (endothelin-1) [25]. This appeared to be due to TNFα-mediated induction of the anti-inflammatory CEACAM1-4L variant in M2 macrophages associated with white adipose tissue to enhance innate immunity [26] and counter inflammation-driven insulin resistance [27].

To address whether endothelial CEACAM1 prevents atherosclerosis, the current study examined whether the insulin sensitive endothelial-*Ceacam1* null mouse on the *Ldlr^−/−^* background (*Ldlr^−/−^VECadCre+Cc1^fl/fl^*) develop atherosclerosis. Because the combined deletion of *Ldlr* and *Ceacam1* is not expected to alter insulin sensitivity [5], this model will predictably test whether hypercholesterolemia causes atherosclerosis in the absence of insulin resistance. 

## 2. Results

### 2.1. Intact Insulin Sensitivity in the Presence of Hypercholesterolemia in Ldlr^−/−^VECadCre+Cc1^fl/fl^ Mice 

Contrary to the insulin-resistant global *BL6.Cc1^−/−^* (*Cc1^−/−^*), *BL6.VECadCre+Cc1^fl/fl^* mice displayed normal insulin metabolism and action [25]. They also showed intact lipid homeostasis at 9 months of age, as assessed by normal hepatic (not shown) and plasma triacylglycerol (Figure S2A(i)), and cholesterol (total, free, VLDL, LDL and HDL) levels compared to their littermate controls (Figure S2A(ii–vi)). Mice with endothelial loss of *Ceacam1* did not develop steatosis in aortae, as revealed by Oil red-O staining of aortic root sections (Figure S2B(i)). Moreover, IHC analysis of aortic roots with CD68 (Figure S2B(ii)) and Trichrome staining (Figure S2B(iii)) revealed no induction in macrophage activation or fibrosis, respectively, in *BL6.VECadCre+Cc1^fl/fl^* nulls relative to their controls. This is in marked contrast to the insulin resistant *Cc1^−/−^* mouse that developed steatosis, inflammation, and fibrosis in aortic roots in the face of limited pro-atherogenic lipidemia when propagated on C57BL6/J background and fed a regular chow diet [17].

**Figure 1:**
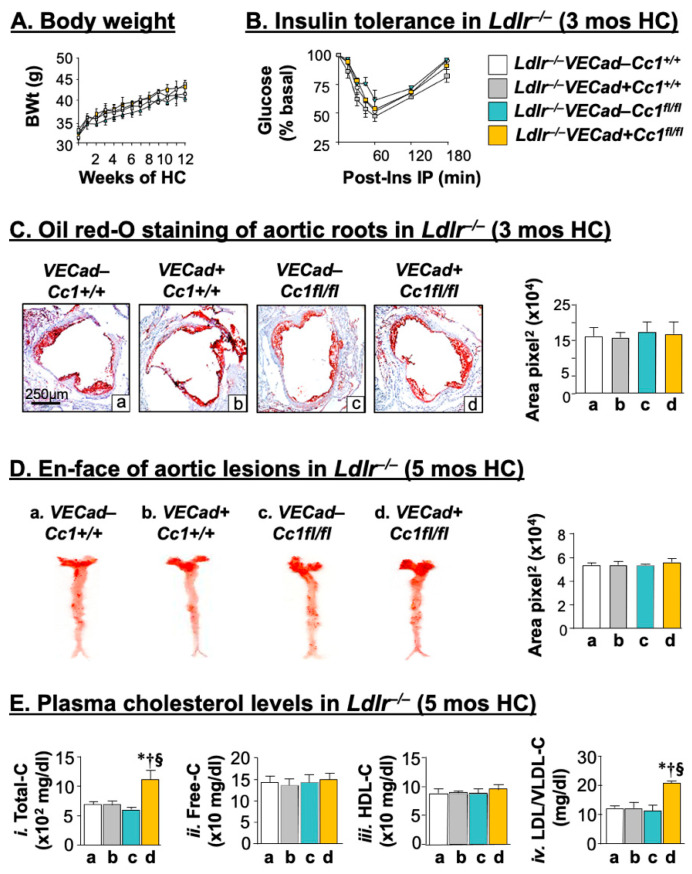
Metabolic phenotyping and morphologic analysis of aortic lesions in mice on the *Ldlr^−/−^* background. *Ldlr^−/−^VECad+Cc1^fl/fl^* mice and littermate controls (n ≥ 5/genotype) were fed HC for 3 months. (**A**) Body weight gain during the feeding period. (**B**) The mice were injected intraperitoneally with insulin (0.75 U/kg BW) to determine glucose clearance 0–180 min post-injection [*Ldlr^−/−^VECad–Cc1^+/+^* (white), *Ldlr^−/−^VECad+Cc1^+/+^* (grey), *Ldlr^−/−^VECad–Cc1^fl/fl^* (teal), and *Ldlr^−/−^VECad+Cc1^fl/fl^* (yellow)]. (**C**) Aortic root sections were analyzed by Oil red-O (ORO) staining and the stained areas measured in pixel^2^ using Image J. Values were expressed as mean ± SEM and presented in the accompanying graph [*Ldlr^−/−^VECad–Cc1^+/+^* (a, white bar), *Ldlr^−/−^VECad+Cc1^+/+^* (b, grey bar), *Ldlr^−/−^VECad–Cc1^fl/fl^* (c, teal bar), and *Ldlr^−/−^VECad+Cc1^fl/fl^* (d, yellow bar)]. (**D**) Atherosclerotic lesions were assessed by ORO-positive lesions on en-face preparation of whole aortae from mice fed HC for 5 months (*n* ≥ 5/genotype). Aortae were dissected from the root to the abdomen and opened longitudinally. Representative en-face views of aortic surface lesions are shown. The extent of atherosclerotic lesion was defined as the percentage of total ORO-positive lesion area over the total surface area, measured in pixel^2^, and values (mean ± SEM) were represented in the accompanying graph [*Ldlr^−/−^VECad–Cc1^+/+^* (a, white bar), *Ldlr^−/−^VECad+Cc1^+/+^* (b, grey bar), *Ldlr^−/−^VECad–*Cc1^fl/fl^ (c, teal bar) and *Ldlr^−/−^VECad+Cc1^fl/fl^* (d, yellow bar)]. (**E**) Retro-orbital venous blood was drawn from overnight-fasted mice (as in D) to assess plasma levels of (i) Total cholesterol (–C), (ii) Free-C, (iii) HDL-C, and (iv) LDL/VLDL-C [*Ldlr^−/−^VECad–Cc1^+/+^* (a, white bar), *Ldlr^−/−^VECad+Cc1^+/+^* (b. grey bar), *Ldlr^−/−^VECad–Cc1^fl/fl^* (c, teal bar), and *Ldlr^−/−^VECad+Cc1^fl/fl^* (d, yellow bar)]. Values were expressed as mean ± SEM. * *p* < 0.05 vs. *Ldlr^−/−^VECad–Cc1^+/+^*; ^†^ *p* < 0.05 vs. *Ldlr^−/−^VECad+Cc1^+/+^*; and ^§^ *p* < 0.05 vs. *Ldlr^−/−^VECad–Cc1^fl/fl^*.

Failure of *BL6.VECadCre+Cc1^fl/fl^* nulls to develop atherogenic-like histological abnormalities could stem from the absence of insulin resistance and pro-atherogenic hypercholesterolemia. To test this hypothesis, we propagated mice on the *Ldlr^−/−^* background and fed them a high-cholesterol (HC) atherogenic diet for 3 months starting at 6 months of age. *Ldlr^−/−^VECadCre+Cc1^fl/fl^* nulls displayed a comparable age-related (Table 1) and diet-induced body weight gain to their littermate controls when fed an HC-rich diet (Figure 1A). This was supported by normal visceral adipose mass in the null mice relative to controls at 9 months of age (Table 1). Steady-state plasma insulin levels remained normal in association with intact insulin secretion (inferred to from intact plasma C-peptide and fasting normoglycemia) and clearance (steady-state C-peptide/insulin molar ratio) (Table 1). Together with normal tolerance to exogenous insulin (Figure 1B) and glucose (not shown), normal fasting insulinemia and random glycemia (Table 1) demonstrated persistent insulin sensitivity in *Ldlr^−/−^VECadCre+Cc1^fl/fl^* mice, as expected of the *Ldlr^−/−^* background, which does not significantly cause/accelerate insulin resistance in the absence of other genetic factors [5].

Consistent with normal visceral adiposity, null mice exhibited intact plasma non-esterified fatty acids (NEFA) (Table 1). They also exhibited intact hepatic and plasma triacylglycerol levels (Table 1). In contrast, plasma LDL cholesterol (LDL-C) and total cholesterol levels were significantly elevated in *Ldlr^−/−^VECadCre+Cc1^fl/fl^* compared to controls (Table 1). This did not derive from changes in cholesterol synthesis, as supported by normal hepatic HMG-CoA reductase (HMGCR) activity in nulls compared to controls (Table 1). It likely derived from reduced LDL clearance as suggested by the higher plasma ApoB100, and proprotein convertase subtilisin/kexin type 9 (PCSK9) levels (Table 1), leading to reduction in LDL-C transport in null mice [28]. Consistently, the mRNA levels of hepatic Pcsk9 were ~2-to-4–fold higher and those of hepatic LDL receptor-related protein 1 (Lrp1) that is involved in hepatocytic clearance of chylomicron remnants were ~2-fold lower in nulls versus controls. Together, this demonstrated that *Ldlr^−/−^VECadCre+Cc1^fl/fl^* developed pro-atherogenic dyslipidemia without insulin resistance. 

### 2.2. Ldlr^−/−^VECadCre+Cc1^fl/fl^ Mice Did Not Develop Atheromatic Plaques 

Oil red-O staining revealed no significant change in fat deposition in the aortic root cross-sections from HC-fed *Ldlr^−/−^VECadCre+Cc1^fl/fl^* mice relative to their littermate controls (Figure 1C). En-face analysis revealed no difference in the fat deposition along the whole aortae of *Ldlr^−/−^VECadCre+Cc1^fl/fl^* and their aged-matched controls even after 5 months of HC intake (Figure 1D and accompanying graph). Moreover, there was no gross aorta dilation or aneurysm formation in null mice. This histology developed in the presence of higher (~2-fold) plasma total–C and LDL–C/VLDL–C levels (Figure 1E) and sustained insulin sensitivity in null mice by comparison to their control littermates (not shown).

### 2.3. Elevated Pro-Inflammatory State in Ldlr^−/−^VECadCre+Cc1^fl/fl^ Aortae

**Figure 2:**
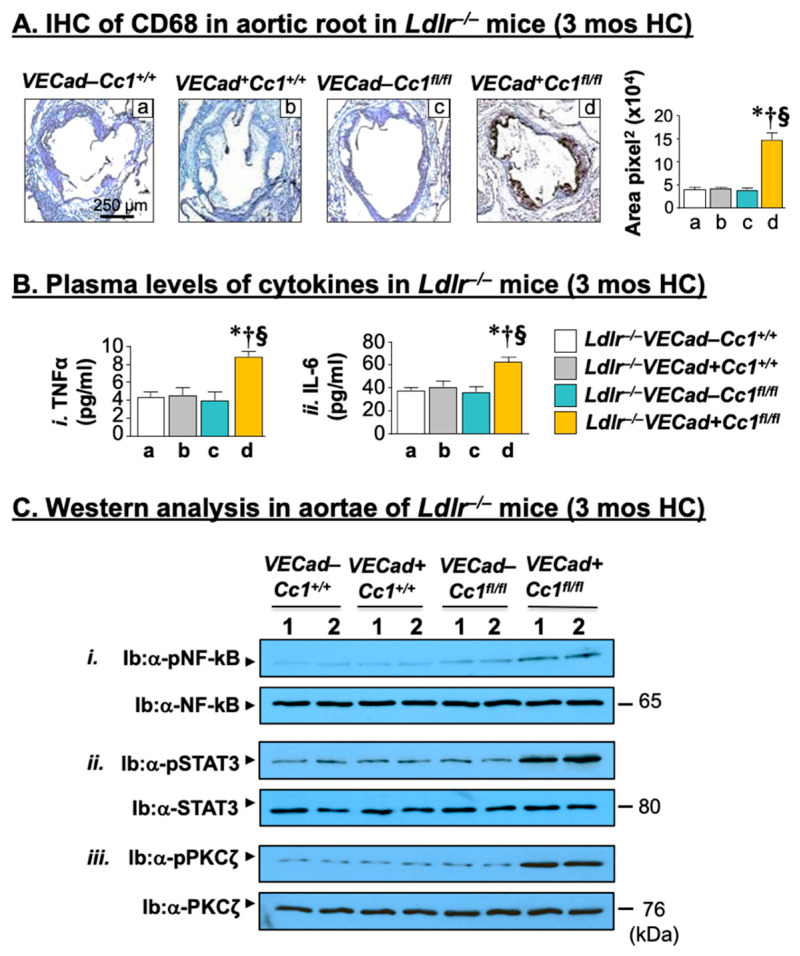
Vascular inflammation. Littermate male mice (n ≥ 5/genotype) were fed HC for 3 months before (**A**) IHC analysis of CD68 in aortic roots was performed. Positive stained areas (brown) calculated as area pixel^2^ using Image J were expressed as mean ± SEM and represented in the accompanying graph [*Ldlr^−/−^VECad–Cc1*^+/+^ (a, white bar), *Ldlr^−/−^VECad+Cc1^+/+^* (b, grey bar), *Ldlr^−/−^VECad–Cc1^fl/fl^* (c, teal bar), and *Ldlr^−/−^VECad+Cc1^fl/^*^fl^ (d, yellow bar)]. * *p* < 0.05 vs. *Ldlr^−/−^VECad–Cc1^+/+^*; ^†^ *p* < 0.05 vs. *Ldlr^−/−^VECad+Cc1^+/+^*; and ^§^ *p* < 0.05 vs. *Ldlr^−/−^VECad–Cc1^fl/fl^*. (**B**) Retro-orbital venous blood was drawn from overnight fasted mice to assess plasma levels of (i) TNFα and (ii) IL-6. The graph represents values as mean ± SEM [*Ldlr^−/−^VECad–Cc1^+/+^* (a, white bar), *Ldlr^−/−^VECad+Cc1^+/+^* (b, grey bar), *Ldlr^−/−^VECad–Cc1^fl/fl^* (c, teal bar), and *Ldlr^−/−^VECad+Cc1^fl/fl^* (d, yellow bar)]. * *p* < 0.05 vs. *Ldlr^−/−^VECad–Cc1^+/+^*; ^†^ *p* < 0.05 vs. *Ldlr^−/−^VECad+Cc1^+/+^*; and ^§^ *p* < 0.05 vs. *Ldlr^−/−^VECad–Cc1^fl/fl^*. (**C**) Western blot analysis was performed on aorta lysates by immunoblotting (Ib) with antibodies (α-) against phosphorylated (i) α-pNF-κB, (ii) α-pSTAT3, and (iii) α-pPKCζ and was normalized by immunoprobing parallel gels with antibodies against total NF-κB, STAT3, and PKCζ, respectively. Numbers (in kDa) to the right of the gels indicate the apparent molecular mass of the signaling proteins. Gels represent analysis on 2 mice/genotype performed on different sets of mice/protein.

Inflammation is a key factor in the development of pro-fibrotic diseases such as atherosclerosis [29]. Consistently, deleting *Ceacam1* from endothelial cells induced the macrophage pool (mRNA of F4/80) (Table S2) and its activation (IHC analysis of CD68-Figure 2A) in *Ldlr^−/−^VECadCre+Cc1^fl/fl^* aortae compared to all three controls. qRT-PCR analysis revealed an increase in the mRNA levels of pro-inflammatory CD4+T and CD8+T cells, but not in the anti-inflammatory Treg pools (Foxp3) in *Ldlr^−/−^VECadCre+Cc1^fl/fl^* aortae (Table S2). 

**Figure 3:**
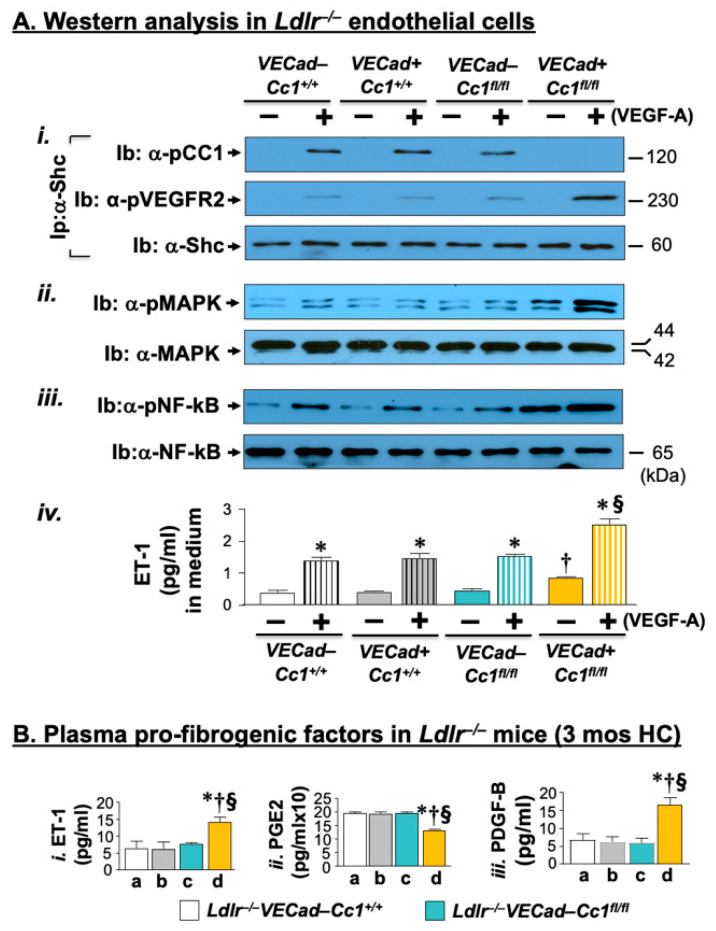
VEGF-A signaling through the Shc/MAP kinase pathway in primary endothelial cells. (**A**) Endothelial cells were isolated from the hearts of 2-month-old mice (*n* ≥ 5/genotype) and treated with (+) or without (–) VEGF-A (40 ng/mL) for 5 min. (i) Lysates were subjected to immunoprecipitation (Ip) with α-Shc antibody followed by immunoprobing with antibodies against Shc, phospho-CEACAM1 (pCC1), and phospho-VEGFR2 (pVEGFR2) to evaluate the level of these proteins in the α-Shc immunopellet. (ii–iii) Lysates were subjected to immunoblotting with α-phospho-antibodies against phosphorylated MAPK and NF-κB and in parallel gels, against MAP Kinase (MAPK), and NF-κB protein for normalization. (iv) Endothelin-1 (ET-1) was assayed in the media of isolated endothelial cells stimulated with VEGF-A for 20 min. Values were expressed as mean ± SEM. * *p* < 0.05 vs. no VEGF-A (solid bars)/same genotype; ^†^ *p* < 0.05 vs. (–) VEGF-A in all 3 controls; ^§^ *p* < 0.05 (+) VEGF-A (vertical lines) in all 3 controls. (**B**) Retro-orbital venous blood was drawn from overnight fasted mice fed HC for 3 months (*n* ≥ 5/genotype) to analyze plasma (i) ET-1, (ii) PGE2, and (iii) PDGF-B. Values were expressed as mean ± SEM. * *p* < 0.05 vs. *Ldlr^−/−^VECad–Cc1^+/+^* (a, white bar); ^†^ *p* < 0.05 vs. *Ldlr^−/−^VECad+Cc1^+/+^* (b, grey bar); and ^§^ *p* < 0.05 vs. *Ldlr^−/−^VECad–Cc1^fl/^*^fl^ (c, teal bar).

Activated macrophages and increased inflammatory response was supported by the higher production of pro-inflammatory cytokines (IL-1β; IL-6; TNFα) (Table S2) and their release into the plasma (Figure 2B). As we have reported in *BL6.VECadCre+Cc1^fl/fl^* mice [25], this could stem from the basal activation (phosphorylation) of their transcriptional master regulator (NF-κB) (Figure 2C(i)). As in response to insulin, VEGF-A, (40 ng/mL, 5 min) induced the pull-down of phosphorylated VEGFR2 in the α-Shc immunopellet in endothelial cells isolated from the hearts of 2-month-old *Ldlr^−/−^VECadCre+Cc1^fl/fl^* mice relative to their control counterparts (Figure 3A(i)). This activated MAP Kinase (Figure 3A(ii)) and NF-κB (Figure 3A(iii)) in null endothelial cells to a higher extent than controls.

As expected from IL-6 activation of STAT3 (signal transducer and activator of transcription 3), *Ldlr^−/−^VECadCre+Cc1^fl/fl^* aortae exhibited higher basal STAT3 phosphorylation (activation) compared to controls (Figure 2C(ii)). Together with NF-κB activation, this induced the mRNA levels of monocyte chemoattractant protein-1 (Mcp-1), Toll-like receptors 2/4 (Tlr-2/4), and the Cd11b+ macrophage pool (Table S2). 

The data demonstrate that null deletion of *Ceacam1* in endothelial cells caused a cell-autonomous positive effect on NF-κB and Stat3 activation and their downstream transcriptional targets to induce an inflammatory microenvironment in *Ldlr^−/−^VECadCre+Cc1^fl/fl^* aortae. 

### 2.4. Increased Leukocyte Adhesion to the Vessel Walls of Ldlr^−/−^VECadCre+Cc1^fl/fl^ Mice

**Figure 4:**
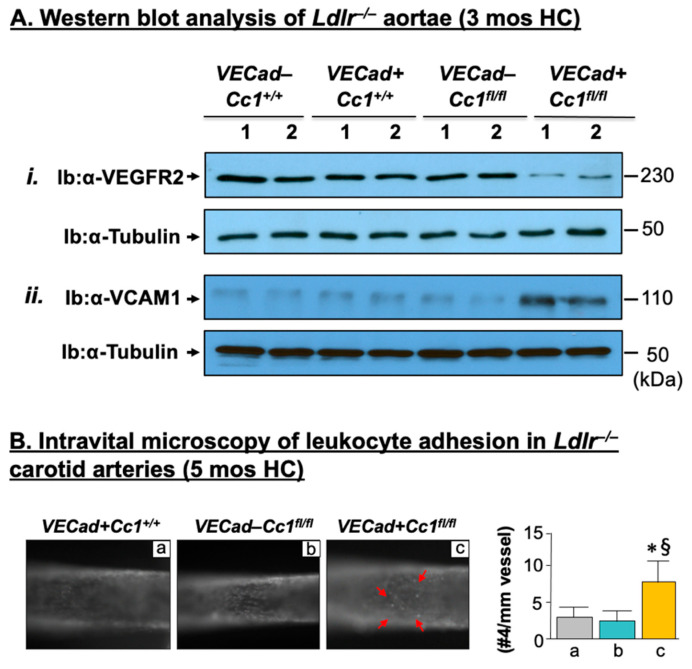
Leukocyte adhesion. (**A**) As in the legend to Figure 2, mice were fed a HC for 3 months and their aorta lysates were subjected to immunoblotting with (i) α-VEGFR2 and (ii) α-VCAM1 followed by probing with α-Tubulin for normalization. Gels represent analysis on 2 mice/genotype performed on different sets of mice/protein. (**B**) Mice (*n* ≥ 5/genotype) were fed a HC for 5 months before their carotid arteries were isolated from the surrounding tissues and intravital microscopy of leukocyte adhesion was assessed, as described in the methods section. Cells that adhered to the vessel wall without rolling or moving for at least 3 sec (red arrows) were counted over the vessel observed by using an intravital microscope. Video images were analyzed offline for leukocyte adhesion. Total numbers were used for statistical analysis. Values were expressed as mean ± SEM and represented in the accompanying graph. * *p* < 0.05 vs. *Ldlr^−/−^VECad+Cc1^+/+^* (a, white bar) and ^§^ *p* < 0.05 vs. *Ldlr^−/−^VECad–Cc1^fl/fl^* (b, grey bar).

Endothelial CEACAM1 prevents vascular permeability by contributing to the regulation of endothelial barrier formation [24]. Accordingly, *Ldlr^−/−^VECadCre+Cc1^fl/fl^* aortae displayed a 2-to-3–fold decrease in the mRNA levels of genes involved in the formation of tight (Zo-1, Claudin1, Claudin5, Occludin) and adherens junctions (VE-Cadherin, β-catenin), which contribute to the maintenance of vascular integrity together with VEGF-A, VEGFR2, VEGFR1 and angiopoietins (Ang-1/2) (Table S3). Western blot analysis confirmed reduction of VEGFR2 in *Ldlr^−/−^VECadCre+Cc1^fl/fl^* aortae relative to controls (Figure 4A(i)). Activation of PKCζ/NF-κB pathways (Figure 2C(iii)) by elevated TNFα could contribute to endothelial cell permeability in *Ldlr^−/−^VECadCre+Cc1^fl/fl^* mice [30].

Consistent with increased inflammation and vascular permeability, deleting *Ceacam1* from endothelial cells induced leukocyte adhesion to the vessel wall of the nulls, as indicated by intravital microscopy of the carotid artery (Figure 4B). This was supported by increased mRNA (Table S2) and protein levels (Figure 4A(ii)) of the vascular cell adhesion molecule (VCAM-1), a transcriptional target of NF-κB and a mediator of leukocyte-endothelial adhesion, as previously shown in bovine aortic endothelial cells with SiRNA-mediated downregulation of Ceacam1 [17]. 

### 2.5. Increased Oxidative Stress in Ldlr^−/−^VECadCre+Cc1^fl/fl^ Mice

Oxidative stress plays a central role in cardiovascular disorders [29]. 3 months of HC induced the mRNA levels of NOX4 and Gp91 in the aortae of *Ldlr^−/−^VECadCre+Cc1^fl/fl^* mice (Table S3). It also increased their plasma NAD/NADH and 8-isoprostane levels with a reciprocal decrease in plasma nitric oxide (NO) levels (Table 1). Moreover, the mRNA level of aortic Niemann-Pick type C1 protein (NPC-1) was significantly reduced in *Ldlr^−/−^VECadCre+Cc1^fl/fl^* mice (Table S3). This was associated with a marked reduction in their plasma GSH levels (Table 1), which could contribute to the robust response to the cytotoxic effects of TNFα. 

**Figure 5:**
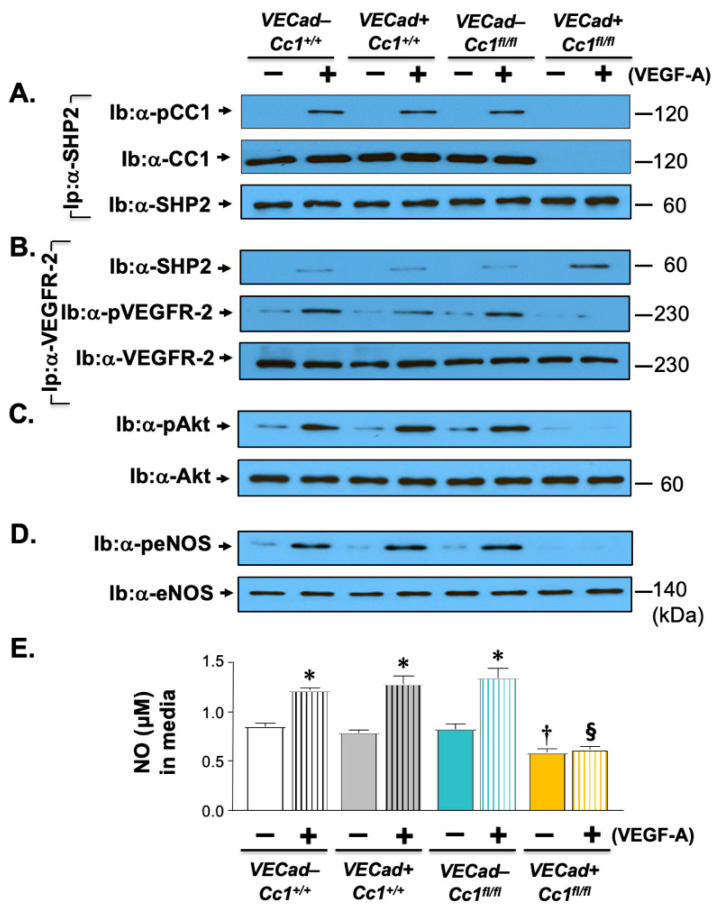
VEGF signaling through the Akt/eNOS pathway in primary endothelial cells. Primary heart endothelial cells were treated with VEGF-A for 5 min, as in the legend of Figure 3. (**A**) Lysates were subjected to immunoprecipitation (Ip) with α-SHP2 antibody followed by immunoblotting (Ib) with antibodies against phospho-CEACAM1 (α-pCC1), CEACAM1 (α-CC1), and α-SHP2 to detect these proteins in the α-SHP2 immunopellet. (**B**) Lysates were immunoprecipitated with the α-VEGFR2 antibody followed by immunoblotting with antibodies against α-SHP2, phospho-VEGFR2, and VEGFR2 to assess the level of these proteins in the VEGFR2 immunopellet. (**C**,**D**) Lysates were subjected to immunoblotting with α-phospho-Akt and α-phospho-eNOS and in parallel gels, against Akt and eNOS, respectively, for normalization. (**E**) Nitric oxide (NO) was essayed in the media of primary heart endothelial cells stimulated with VEGF-A (40 ng/mL) for 20 min. Values were expressed as mean ± SEM. * *p* < 0.05 vs. no VEGF-A (solid bars)/same genotype; ^†^ *p* < 0.05 vs. (−) VEGF-A in all 3 controls; ^§^ *p* < 0.05 (+) VEGF-A (vertical lines) in all 3 controls.

Inflammation and oxidative stress mediate endothelial injury resulting from low NO bioavailability [4]. Consistently, 3 months of HC lowered aortic NO levels in *Ldlr^−/−^VECadCre+Cc1^fl/fl^* mice (Table 1). As in insulin treatment of endothelial cells from *BL6.VECadCre+Cc1^fl/fl^* mice [25], VEGF-A treatment induced binding of SHP2 to VEGFR2 (Figure 5B) in the absence of its sequestration by CEACAM1 in *Ldlr^−/−^VECadCre+Cc1^fl/fl^* endothelial cells (Figure 5A). Consequently, phosphorylation of VEGFR2 (Figure 5B) and of its Akt/eNOS downstream signaling pathway (Figure 5C,D) was reduced, stemming NO production and its release from null endothelial cells (Figure 5E). 

### 2.6. Increased Fibrosis in Ldlr^−/−^VECadCre+Cc1^fl/fl^ Aortic Roots 

**Figure 6:**
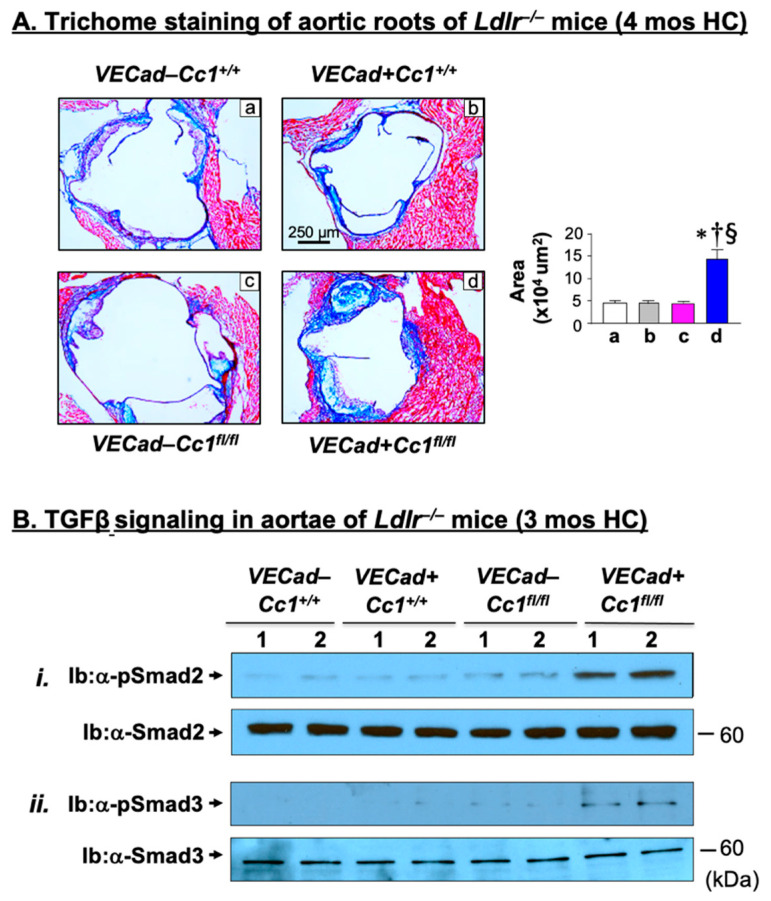
Vascular fibrosis. Male mice (n = 4/genotype) were fed HC for 4 months. (**A**) Aortic roots were stained with Gomori trichrome to detect collagen deposition. The total positively stained areas (blue) were calculated as area pixel^2^ using Image J and are presented in the accompanying graph. * *p* < 0.05 vs. *Ldlr^−/−^VECad–Cc1^+/+^* (a, white bar); ^†^ *p* < 0.05 vs. *Ldlr^−/−^VECad+Cc1^+/+^* (b, grey bar) and ^§^ *p* < 0.05 vs. *Ldlr^−/−^VECad–Cc1^fl/fl^* (c, teal bar). (**B**) Aortic lysates from mice fed HC for 3 months were analyzed by immunoblotting with (i) α-phospho-Smad2 (α-pSmad2) and (ii) α-phospho-Smad3 (α-pSmad3) antibodies and with antibodies against Smad2 and Smad3, respectively, in parallel gels for normalization. Gels represent analysis on 2 mice/genotype performed on different sets of mice/protein.

Gomori trichrome staining indicated higher accumulation of fibrotic fibers in the sub-endothelium of the aortic roots of *Ldlr^−/−^VECadCre+Cc1^fl^*^/fl^ mice compared with their age-matched littermate controls after 4 months (Figure 6A and accompanying graph), but not after 3 months (not shown) of HC feeding. Moreover, qRT-PCR analysis revealed a 2-to-5-fold increase in the aortic mRNA levels of pro-fibrogenic genes in *Ldlr^−/−^VECadCre+Cc1^fl/fl^* mice relative to controls: fibronectin, Ctgf, α-Sma, collagen6α3, and Tgfβ (Table S3). Consistent with the ~3-fold decrease in the mRNA level of Smad7 (Table S3), an inhibitor of the TGFβ–Smad2/3 canonical pathway, this pro-fibrogenic pathway was more highly activated (phosphorylated) in *Ldlr^−/−^VECadCre+Cc1^fl/fl^* aortae relative to controls (Figure 6B). 

Elevated fibrogenesis in *Ldlr^−/−^VECadCre+Cc1^fl/fl^* aortae could be mediated by increased NF-κB–mediated transcription of endothelin-1 (ET-1) and of its receptor-A (Etar) (Table S3), which transmits its pro-fibrogenic activity [31]. Given that ET-1 is predominantly produced in endothelial cells, its release from the endothelial cells of nulls (Figure 3A(iv)) likely played a critical role in the ~2-fold higher ET-1 plasma levels in *Ldlr^−/−^VECadCre+Cc1^fl/fl^* mice compared to controls (Figure 3B(i)). Increased plasma levels of ET-1 could result in part from lower plasma PGE2 (Figure 3B(ii)) levels in null mice [32]. In addition to ET-1, activated NF-κB stimulated the transcription of PDGF-B (Figure 3B(iii)) to synergize with ET-1 to mediate fibrogenesis in *Ldlr^−/−^VECadCre+Cc1^fl/fl^* aortae [31]. 

### 2.7. Increased Inflammation and Fibrosis in the Livers of Ldlr^−/−^VECadCre+Cc1^fl/fl^ Mice 

**Figure 7:**
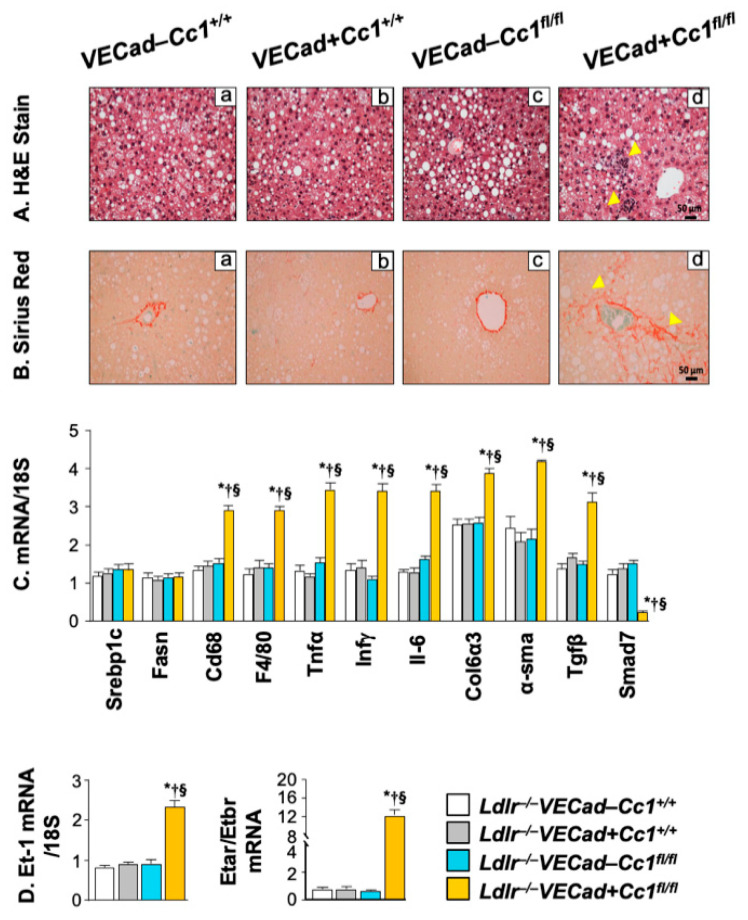
Hepatic inflammation and fibrosis analysis. Livers of littermate mice (n ≥ 5/genotype) fed with HC for 3 months and described in the legend of Figure 2 were extracted and subjected to: (**A**) H&E for histological analysis and (**B**) Sirius Red staining to detect parenchymal inflammatory infiltration and chicken-wire bridging fibrosis (yellow arrowheads), respectively, in *Ldlr^−/−^VECad+Cc1^fl/fl^* mice (panel d) but not in their three controls (panel a–c). (**C**,**D**) qRT-PCR analysis (normalized to 18S) of mRNA levels of hepatic genes involved in lipogenesis, inflammation, and fibrosis. Values were expressed as mean ± SEM. * *p* < 0.05 vs. *Ldlr^−/−^VECad–Cc1^+/+^,* ^†^ *p* < 0.05 vs. *Ldlr^−/−^VECad+Cc1^+/+^*, ^§^ *p* < 0.05 vs. *Ldlr^−/−^VECad–Cc1^fl/fl^*.

Disruption of the integrity of endothelial cells, the second largest cell population in the liver, facilitates the recruitment of neutrophils to contribute to hepatic fibrosis [33,34]. Thus, we next investigated whether endothelial *Ceacam1* deletion altered liver histology. H&E analysis of liver sections from mice fed HC for 3 months revealed extensive parenchymal inflammatory foci formation (arrowheads) without an increase in steatosis in *Ldlr^−/−^VECadCre+Cc1^fl/fl^* mice compared to controls (Figure 7A panels d vs. a–c). Like global *Cc1^−/−^* mice [20], this occurred without ballooning or changes in hepatocellular architecture. Consistently, hepatic mRNA of lipogenic genes such as Srebp-1c and fatty acid synthase (Fasn) (Figure 7C) and hepatic triacylglycerol levels and HMGCR activity were normal (Table 1). 

As in aortae, the macrophage pool in *Ldlr^−/−^VECadCre+Cc1^fl/fl^* livers was induced and activated, as assessed by higher F4/80 and CD68 hepatic levels, respectively (Figure 7C). In addition to activated endothelial cells, this could contribute to increased hepatic mRNA levels of pro-inflammatory genes (IL-1β; IL-6; TNFα). In addition, hepatic mRNA levels of IFNγ points to increased activation of lymphocytes in null versus control mice (Figure 7C). Consistent with higher TNFα, hepatic Smad7 mRNA levels were lower in *Ldlr^−/−^VECadCre+Cc1^fl/fl^* livers (Figure 7C). Together with their higher hepatic Tgfβ mRNA levels, this suggests increased activation of the TGFβ signaling pathway to mediate hepatic fibrosis. Hepatic fibrosis was demonstrated by increased mRNA levels of α-SMA and Col6α3 pro-fibrogenic genes (Figure 7C) and manifested by increased interstitial chicken-wire bridging pattern of collagen deposition in Sirius Red-stained *Ldlr^−/−^VECadCre+Cc1^flfl^* liver sections (Figure 7B panel d vs. a–c). Consistently, the hepatic mRNA levels of ET-1 and in its pro-fibrogenic ET_A_R receptors were induced with a reciprocal decrease in the anti-fibrogenic ET_B_R mRNA levels in *Ldlr^−/−^VECadCre+Cc1^flfl^* livers compared to control mice (Figure 7D). This could be partly caused by hyperactivation of NF-κB in endothelial cells in response to their loss of *Ceacam1* gene [25,35]. 

## 3. Discussion

Dissecting out the role of altered metabolism [dyslipidemia and insulin resistance] from endothelial cell injury in the pathogenesis of atherosclerosis is challenging [36], in part owing to the causal role that dyslipidemia can play in endothelial cell injury [4]. The current studies revealed that feeding the insulin sensitive *Ldlr^−/−^VECadCre+Cc1^fl/fl^* mice with a high cholesterol diet for 3–5 months caused hypercholesterolemia (mostly LDL-C) without insulin resistance, visceral obesity or increase in plasma NEFA and other dyslipidemia-related changes. Interestingly, no atherosclerotic plaques developed in *Ldlr^−/−^VECadCre+Cc1^fl/fl^* mice, and their histological abnormalities were limited to inflammation and fibrosis in the aortae without fatty streaks. A similar phenotype developed in the livers of these mice in the absence of higher hepatic steatosis than that found in control mice. In contrast, hepatocyte-specific *Ceacam1* deletion caused overt atheroma plaque formation and NASH in the presence of insulin resistance [21]. Together, this points to a key role for systemic insulin resistance and its associated metabolic derangements, such as visceral obesity, lipolysis and hepatic steatosis, in plaque formation in aortae. 

Hypercholesterolemia in *Ldlr^−/−^VECadCre+Cc1^fl/fl^* mice was marked by higher LDL-C levels likely resulting from reduced clearance instead of synthesis. Despite the adverse effect of CEACAM1 deletion on endothelial cell integrity and increased cell permeability in *Ldlr^−/−^VECadCre+Cc1^fl/fl^* mice, consistent with previous reports [24], possible LDL-C trans-endothelial transport and redistribution to the subendothelial intima did not appear to occur to sufficient levels to result in fatty streaks. This provides in vivo evidence that fatty streaks do not advance to plaque formation in the absence of insulin resistance. 

In addition to dyslipidemia, the pro-inflammatory state brought about by increased visceral obesity links endothelial cell injury and dysfunction to early atherosclerosis [4]. As with individual deletion of endothelial *Ceacam1* [25], combined deletion with *Ldlr* caused NF-kB driven increases of inflammatory cytokines (such as TNFα and IL-6) in endothelial cells and release into the plasma without causing visceral obesity and insulin resistance (also marked by normal NEFA levels). The increase in endothelial TNFα may contribute to increased endothelial cell permeability [37] and disruption of the endothelial cell barrier [38] and subsequently, to the development of early atherosclerosis in *Ldlr^−/−^VECadCre+Cc1^fl/fl^* mice. 

Insulin resistance in endothelial cells plays an important role in atherosclerosis by reducing insulin signaling through the Akt/eNOS pathway [39]. This does not only reduce NO production and cause oxidative stress, but also reduces endothelin-1B receptor expression to mediate atherosclerosis [40]. Consistently, endothelial cell-specific *Ceacam1* deletion reduced Akt/eNOS activation by VEGF-A, leading to reduced VEGFA-stimulated NO production, endothelin-1B receptor expression, redox imbalance and oxidative stress in double *Ceacam1*/*Ldlr* knockouts. As in response to insulin [25], a compromised Akt/eNOS pathway in endothelial cells was accompanied by an increased Shc/NF-kB–dependent pathway and VEGFA-stimulated endothelin-1 production. This induced aortic inflammation and fibrosis [in part by activating TGFβ pathways [41], consistent with endothelial gain-of-function of endothelin 1 [42] and reduced NO production [43]. 

In contrast to endothelial cells, deleting *Ceacam1* in hepatocytes caused hyperinsulinemia-driven systemic insulin resistance and visceral obesity associated with features of non-alcoholic fatty liver disease and atherosclerosis when mice were backcrossed on the *Ldlr* null background and fed an atherogenic diet for 3 months [21]. These mice developed hypercholesterolemia due to increased cholesterol synthesis. Histopathologically, mice developed aortic plaques with lipid accumulation, inflammation and fibrosis [21]. The phenotype of mice with hepatocyte-specific deletion of *Ceacam1* faithfully replicated the human disease in contrast to that of *Ldlr^−/−^VECadCre+Cc1^fl/fl^* mice in which the metabolic derangement was restricted to elevation in total and LDL cholesterol without insulin resistance or visceral obesity. This points to targeting insulin resistance as an effective preventive/treatment measure against atherosclerosis. With insulin resistance constituting an independent predictor of endothelial dysfunction in individuals without classical risk factors for atherosclerosis [6], insulin sensitizers are increasingly being considered as a promising preventive approach [8].

Thus, endothelial cell-specific deletion of *Ceacam1* on *Ldlr* null background caused hypercholesterolemia without insulin resistance. It also caused vascular inflammation and fibrosis without increased atheroma formation relative to controls. Mechanistically, this was underlined by reduced NO bioavailability and increased oxidative stress driven by blunted Akt/eNOS pathway in response to VEGF, as we previously reported for insulin [25]. This endothelial loss of *Ceacam1* also caused hyperactivation of Shc/NF-kB signaling and its downstream pro-inflammatory and endothelin 1-dependent pro-fibrogenic pathways. Because CEACAM1 is a shared element between insulin and VEGF-A signaling, the data showed that altered signaling through CEACAM1-dependent pathways disrupts endothelial cells’ response to insulin and VEGF-A, and in this cell-autonomous fashion, drives profound endothelial dysfunction, which in the absence of insulin resistance did not advance to overt atheroma formation.

In summary, the current studies showed that, in the absence of insulin resistance, hypercholesterolemia did not lead to overt atheroma formation or hepatic steatosis despite increased development of inflammation and fibrosis along the aortic wall and in the liver. This supports a critical role for hyperinsulinemia and insulin resistance in the pathogenesis of atherosclerosis and hepatic steatosis.

Research limitations and future perspectives: The current studies showed that despite increased LDL-C levels and its trans-endothelial transport through the endothelial cell that was rendered permeable by the loss of *Ceacam1*, fatty streaks did not develop on the aortic walls of *Ldlr^−/−^VECadCre+Cc1^fl/fl^* mice. While it is possible that a much higher level of LDL-C is needed to cause fatty streaks in mice as compared to humans, these observations assign a stronger role for insulin sensitizers in the prevention of atherosclerosis. This would be consistent with outcome studies showing that lowering plasma cholesterol levels does not suffice to stop the progression of atherosclerosis in patients with metabolic disease [44]. It also supports the observation that pioglitazone, an insulin sensitizer and a PPARγ agonist, ameliorates atherosclerosis in at risk patients [8]. Whether this drug can prevent atherosclerosis by inducing Ceacam1 transcription [45] in endothelial cells and hepatocytes of patients with cardiovascular risk remains to be tested. 

## 4. Materials and Methods

### 4.1. Generation of Null Mice

*C57BL6/J.VECadherinCre+Cc1^fl/fl^* mice (*BL6.VECadCre*+*Cc1^fl/fl^* or *VECad+Cc1^fl/fl^*) were generated using transgenic Cre mice under the transcriptional control of the VECadherin promoter (*VECadherin Cre*), as described previously [25]. Mice were then backcrossed with the low-density lipoprotein receptor deficient mouse *C57BL6/J.Ldlr^−/−^* (termed *Ldlr^−/−^* for simplicity) (Jackson Laboratories, Bar Harbor, ME, USA) for ≥ 6 generations to generate four homozygous littermates: *Ldlr^−/−^VECadCre–Cc1^+/+^* (WT); *Ldlr^−/−^VECadCre+Cc1^+/+^* (Cre control); *Ldlr^−/−^VECadCre–Cc1^fl/fl^* (Flox control); and *Ldlr^−/−^VECadCre+Cc1^fl/fl^* (null mice). Genotyping analysis is described in more details in Supplementary Materials. Of note, the *Ldlr* null model was used instead of the *ApoE* null because the latter locus is located on murine chromosome 7 in close proximity to that of *Ceacam1*, preventing recombination of both alleles. 

Animals were housed in a 12-h dark-light cycle and fed a standard regular chow diet until 6 months of age when male mice were fed *ad libitum* a high cholesterol atherogenic diet (HC) of 42% kcal from fat, 0.2% total cholesterol, and 2.7% kcal from carbohydrate (high sucrose 34% by weight) (Harlan Teklad, TD.88137, Haslett, MI, USA) for 3–5 months. Body weight was assessed weekly. Of note, the age and the duration of feeding were optimized from several cohorts of mice that were fed HC diet for different periods of time and starting at different ages. Feeding HC for 3 months was found to be the shortest period to cause altered histology in the aortae of null mice. All procedures and animal experiments were approved by the Institutional Animal Care and Utilization Committee in all participating institutions.

### 4.2. Tolerance Tests

Mice were fasted from 7:00 a.m. until 1:00 p.m. before being injected intraperitoneally (IP) with insulin (0.75 U/kg BW, Novo Nordisk, Princeton, NJ, USA). Tail blood was then collected to assess glucose levels at 0–180 min post-injection, as routinely done [25]. 

### 4.3. Plasma and Tissue Biochemistry

Mice were fasted from 5:00 p.m. until 11:00 a.m. the following morning and retro-orbital blood was drawn to assess plasma levels of: free, total, LDL-VLDL, and HDL cholesterol (using Cholesterol assay kit, ab65390, Abcam, Cambridge, MA, USA); LDL (Mouse LDL-Cholesterol kit, 79980, Crystal chem, Elk Grove Village, IL, USA); VLDL [calculated by dividing plasma triacylglycerol (Pointe Scientific, Canton, MA, USA) by 5]; ApoB100 and ApoB48 (MyBiosource ELISA kits, MBS2502404 and MBS744267, respectively, San Diego, CA); PCSK9 (PCSK9 ELISA kit, ab215538, Abcam, Cambridge, UK); nitric oxide (NO) (Nitrate/Nitrite Fluorometric Assay Kit, 780051, Cayman Chemical, Ann Arbor, MI, USA); NAD/NADH (Total NAD and NADH colorimetric Assay Kit, ab186032, Abcam, Cambridge, UK); Glutathione (GSH) (Glutathione detection assay kit, ab65322, Abcam); 8-isoprostane (8-isoprostane ELISA kit, ab175819, Abcam, Cambridge, UK); TNFα (SimpleStep ELISA kit, ab208348, Abcam, Cambridge, UK); IL-6 (Interleukin-6 ELISA Kit, ab222503, Abcam, Cambridge, UK); endothelin-1 (Endothelin-1 ELISA kit, ab133030, Abcam); prostaglandin E2 (prostaglandin E2 ELISA kit, ab133021, Abcam, Cambridge, UK); PDGF-B (ELISA Kit, MBB00; R&D System, Minneapolis, MN, USA). Hepatic triacylglycerol was determined as previously described [46] and aortic NO content as described above for plasma levels. Hepatic HMG-CoA Reductase (β-hydroxy β-methylglutaryl coenzyme A) activity was assayed in homogenized tissues per manufacturer’s instructions (HMG-CoA Reductase Activity Colorimetric Assay Kit, ab204701, Abcam, Cambridge, UK).

### 4.4. Isolation of Primary Cells

Primary heart endothelial cells were isolated from ketamine/xylazine-anesthetized 2-to-3–month-old mice, as previously described [25]. Briefly, the heart was Collagenase A-digested and cells were sorted by anti-Mouse CD31, immobilized on Sheep anti-Rat IgG Dynabeads, and cultured on 0.1% gelatin pre-coated plates in growth medium [DMEM (Gibco Lab., Gaithersburg, MD, USA), 0.01% heparin, 10% Bovine Endothelial Cell Growth Supplement-ECGS (Cell Applications, Inc-San Diego, CA, USA), 20% FBS, 1% Penicillin–Streptomycin] for ~3 weeks until they reach ~80% confluency. Cells were then seeded at a density of 3 × 10^5^ cells/well in 6-well plates and allowed to grow for 24 h before being serum-starved for 2 h in phenol red-free medium (Invitrogen, Waltham, MA, USA) supplemented with 25 mM HEPES and 0.1% BSA before being incubated in the presence or absence of 40 ng/mL VEGF-A (R&D systems, Minneapolis, MN, USA) for 5 or 20 min for Western blot and media analysis, respectively. 

### 4.5. Aortic Root Sectioning and Plaque Analysis

As previously described [21], hearts were perfused through the left ventricle with 1× phosphate buffered saline (PBS, Thermo Scientific, Waltham, MA, USA), followed by 4% paraformaldehyde (Sigma-Aldrich, St. Louis, MO, USA), cut and embedded in optimal cutting temperature compound (OCT, Tissue-Tek, 4583, Torrance, CA, USA). This was followed by frozen-sectioning on a microtome-cryostat (10 µm) starting from where the aorta exits the ventricle and moving towards the aortic sinus. Sections were stained with ORO or trichrome (Gomori’s Trichrome Stain Kit, 87020, Thermo Scientific, Walthman, MA, USA), or subjected to immunohistochemical (IHC) analysis with CD68 antibody (1:300; MCA1957GA, Bio-Rad, Hercules, CA, USA) for 3 h at room temperature prior to incubating with a biotinylated goat anti-rat antibody (BA-9401, Vector laboratories, Burlingame, CA, USA) for 1 h. Images were taken at 4X using an Olympus SZX7-TR30 microscope (Tokyo, Japan) and quantified CellSens Standard program.

### 4.6. En-Face Oil-Red-O Staining

As in [21], aortae were isolated starting from the aortic arch down to the femoral arteries and fixed in 10% formalin for 24 h. Longitudinally-opened aortae were cleaned from fat and surrounding connective tissues under a dissection microscope, rinsed with water and 60% isopropanol before undergoing staining with Oil Red-O (ORO) solution (Sigma-Aldrich) for 15 min, rinsed again, mounted to slide glass with lumen down, fixed with a cover slip and scanned with Olympus CKX41. Atherosclerotic lesions were defined as the percentage of total ORO-positive area/total surface area, determined by CellSens Standard software in area pixel^2^.

### 4.7. Intravital Microscopy of Leukocyte Adhesion in Carotid Artery

Following sedation with ketamine and xylazine (100/10 mg/kg), mice were fixed on a 15-cm cell-culture lid in a supine position. As previously described [47], the right jugular vein and left carotid artery were exposed and injected with 100 µL of 0.5 mg/mL rhodamine 6G (R4127; Sigma-Aldrich, St. Louis, MO, USA) to label cells, including leukocytes, with mitochondria. A U-shaped black plastic was placed under the carotid artery (~4–5 mm) to block background fluorescence during real life observation using an intravital microscope (Leica DM6 FS, Buffalo Grove, IL, USA). Video images were captured with a color digital camera (Teledyne QImaging, Surrey, BC, Canada) and analyzed offline for leukocyte adhesion. Cells that adhered to the vessel wall without rolling or moving for at least 3 s were counted over the vessel observed. Total numbers were used for statistical analysis.

### 4.8. Liver Histology

As previously described [21], formalin-fixed, paraffin-embedded sections were stained with hematoxylin-eosin (H&E). Liver sections were deparaffinized and rehydrated before being stained with 0.1% Sirius Red stain (Direct Red80, Sigma-Aldrich, MO, USA). 

### 4.9. Western Blot Analysis

Tissues and cells were lysed and proteins analyzed by SDS-PAGE followed by immunoprobing with polyclonal antibodies (Cell signaling, Danvers, MA, USA) against: phospho-Akt (Ser473), Akt, phospho-p44/42 MAPK (Thr202/Tyr204), p44/42 MAPK, phospho-eNOS (Ser1177), eNOS, phospho-Smad2Ser465/467, Smad2, phospho-Smad3 (Ser423/425), Smad3, phospho-Stat3 (Tyr705), Stat3, phospho-NF-ϏB (Ser536), NF-ϏB, phospho-PKCζ (phospho-Thr410/403), PKCζ, phospho-VEGFR2 (Try1175), VEGF-R2, VCAM1 and Tubulin. A custom-made rabbit polyclonal antibody (Ab 3759) were used against mouse CEACAM1 extracellular domain and phospho-CEACAM1 (α-pCC1) (Bethyl Laboratories, Montgomery, TX, USA). Blots were incubated with horseradish peroxidase-conjugated donkey anti-rabbit IgG antibody (GE Healthcare Life Sciences, Amersham, Marlborough, MA, USA) and proteins were visualized using ECL (Amersham). Polyclonal antibodies against SHP2, and Shc (Cell signaling) were used in co-immunoprecipitation experiments. These membranes were incubated with light chain specific horseradish peroxidase-conjugated IgG monoclonal mouse anti-rabbit (211-032- 171, Jackson Immuno-Research laboratories) to prevent detection of the antibody heavy chain. 

### 4.10. Real-Time Quantitative RT-PCR

Total RNA was isolated from aortae with RNeasy fibrous tissue kit (74704, QIAGEN, Germantown, MD, USA). cDNA was synthesized by iScript cDNA Synthesis Kit (Bio-Rad, Hercules, CA, USA), using 1 μg of total RNA and oligodT primers. qRT-PCR was performed using Fast SYBR Green Master Mix by the ABI StepOnePlus Real-Time PCR System (Applied Biosystems, Beverly, MA, USA), as described [46]. All primers (Table S1) were used at a final concentration of 10 µM. mRNA was normalized relative to ribosomal 18S. 

### 4.11. Statistical Analysis

Data were analyzed by one-way analysis of variance (ANOVA) with Tukey’s for multiple comparisons using GraphPad Prism 7 software. *p* < 0.05 was considered statistically significant.

## Figures and Tables

**Table 1:** Plasma and tissue biochemistry in 9-month-old mice propagated on *Ldlr^−/−^* background and fed an atherogenic high cholesterol diet in the last 3 months.

	*Ldlr^−/−^* *VECad–Cc1^+/+^*	*Ldlr^−/−^* *VECad+Cc1^+/+^*	*Ldlr^−/−^* *VECad–Cc1^fl/fl^*	*Ldlr^−/−^* *VECad+Cc1^fl/fl^*
Body weight (g)	41.5 ± 1.9	43.8 ± 1.9	40.0 ± 1.4	42.1 ± 1.8
% Visceral fat (WAT/BW)	4.6 ± 0.5	4.3 ± 0.5	3.9 ± 0.5	4.2 ± 0.2
Fasting blood glucose (mg/dL)	110 ± 9	108 ± 7	115 ± 15	104 ± 8
Fed blood glucose (mg/dL)	120 ± 7	108 ± 9	116 ± 5	132 ± 5
Plasma Insulin (pM)	23 ± 4	19 ± 1	19 ± 3	23 ± 2
Plasma C-peptide (pM)	377 ± 47	342 ± 29	343 ± 39	341 ± 29
Plasma C/I molar ratio	11 ± 4	12 ± 2	14 ± 2	12 ± 2
**Hepatic lipid**				
Triacylglycerol (µg/mg protein)	86 ± 11	83 ± 5	80 ± 6	87 ± 3
HMGCR (U/mg protein × 10^3^)	22 ± 1	22 ± 1	21 ± 3	23 ± 1
**Plasma lipid**				
NEFA (mEq/L × 10^–2^)	28 ± 3	28 ± 1	28 ± 3	25 ± 2
Triacylglycerol (mg/dL)	69 ± 2	67 ± 6	69 ± 3	67 ± 6
Total Cholesterol (mg/dL)	685 ± 26	686 ± 36	596 ± 15	1043 ± 38 * ^† §^
Free Cholesterol (mg/dL)	151 ± 8	155 ± 8	149 ± 4	152 ± 13
VLDL-C (mg/dL)	130 ± 1	130 ± 3	135 ± 1	130 ± 1
LDL-C (mg/dL)	184 ± 3	169 ± 9	179 ± 9	234 ± 3 * ^† §^
HDL-C (mg/dL)	85 ± 3	87 ± 3	85 ± 3	88 ± 4
ApoB100 (ng/mL)	368 ± 25	455 ± 62	395 ± 63	1872 ± 137 * ^† §^
ApoB48 (µg/mL)	1907 ± 35	1765 ± 50	1828 ± 102	1790 ± 81
PCSK9 (pg/mL)	262 ± 8	241 ± 11	251 ± 8	338 ± 3 * ^† §^
**Plasma redox parameters**				
NO (µM × 10^−1^)	6.3 ± 0.1	6.3 ± 0.3	6.4 ± 0.6	3.9 ± 0.2 * ^† §^
NAD/NADH (µM)	4.9 ± 0.1	4.4 ± 0.8	3.6 ± 0.4	7.7 ± 0.7 * ^† §^
8-isoprostane (pg/mL × 10)	8.8 ± 1.1	9.1 ± 1.0	7.6 ± 1.1	15.0 ± 1.3 * ^† §^
GSH (µg/mL × 10^−1^)	11.2 ± 0.8	15.1 ± 0.5	19.2 ± 0.6	4.9 ± 1.7 * ^† §^
**Aortic NO (µM/µg × 10^−2^)**	51.5 ± 1.1	53.4 ± 1.1	52.5 ± 2.1	19.3 ± 1.0 * ^† §^

Except for andom blood glucose level that was assessed in blood drawn at 10:00 pm, male mice (*n* > 7/genotype) were fasted from 5:00 pm until 11:00 am in the morning of the following day. Retro-orbital blood was drawn, and tissues were collected. Visceral adiposity: % of gonadal plus inguinal white adipose tissue per body weight; the steady-state plasma C-peptide/insulin (C/I) molar ratio was calculated as a measure of insulin clearance; NEFA: non-esterified fatty acids. Plasma VLDL-C was calculated as triacylglycerolx0.2. Data were analyzed by one-way ANOVA with Tukey’s for multiple comparisons and values were expressed as mean ± SEM. * *p* < 0.05 vs. *Ldlr^−/−^VECad–Cc1^+/+^*, ^†^ *p* < 0.05 vs. *Ldlr^−/−^VECad+Cc1^+/+^*, ^§^ *p* < 0.05 vs. *Ldlr^−/−^VECad–Cc1^fl/fl^*.

## Data Availability

The data presented in this study are available on request from the corresponding author.

## Supplementary Materials

The following supporting information can be downloaded at: https://www.mdpi.com/article/10.3390/ijms23084335/s1.
