# Supplementary material for: Aortic Fibrosis in Insulin-Sensitive Mice with Endothelial Cell-Specific Deletion of Ceacam1 Gene
Source: Int J Mol Sci. 2022 Apr 14;23(8):4335. doi: 10.3390/ijms23084335 (PMC9027102; doi:10.3390/ijms23084335)
Supplement: Supplementary file 1 [file ijms-23-04335-s001.zip › ijms-1624039-supplementary.pdf]

International Journal of *Molecular Sciences*

MDPI

Supplementary Materials

Materials and Methods

Genotyping of mice

*C57BL/6J.VECadherinCre+Cc1^fl/fl^* (*VECadCre+Cc1^fl/fl^*) mice were generated and the specific deletion of both of the short and long isoforms of Ceacam1 was confirmed in isolated endothelial cells as described previously [25]. Mice were backcrossed with C57BL/6J.*Ldlr*$^{-/-}$ mice (Jackson Laboratories, Bar Harbor, ME) for ≥6 times. PCR analysis of ear DNA was employed to genotype offsprings using *Ceacam1 (Cc1)*-specific primers (Fig. S1) and identify *ldlr*$^{-/-}$ homozygous mice with wild-type (WT) *Cc1* allele with *VECadCre* (*ldlr*$^{-/-}$*VECadCre+Cc1*$^{+/+}$) (Cre controls) or without (*ldlr*$^{-/-}$*VECadCre–Cc1*$^{+/+}$) (WT controls), and with *Cc1*-floxed allele with *VECadCre* (*ldlr*$^{-/-}$*VECadCre+Cc1*$^{fl/fl}$) (null mice) or without (*ldlr*$^{-/-}$*VECadCre–Cc1*$^{fl/fl}$) (Flox controls). All lines were from the same breeding to limit the confounding effects of LoxP and *VECadherin*.

As previously described [25], the Flox gene was detected by a PCR reaction using FloxA forward primer (FP) with FloxB and FloxC reverse primers (RP) (Fig. S1). The FloxA/FloxB primer set detected the 382bp wild-type allele and the FloxA/FloxC set detected the 488bp Flox allele. As expected, a 488bp sequence from the Flox gene was only amplified in mice positive for *Cc1*$^{flox/flox}$, while a 382bp product was only detected in mice positive for wild-type allele.

Using the Cre primer sets, we detected *VECadCre+* allele (300bp *VECadherin* promoter-Cre) and the *VECadherin* gene only (550bp for *VECadCre–* allele).

To identify the *Ldlr–/–* mutant band of 350bp, an additional reaction was performed using the OMIR33 49 primer forward and the primer reverse pairs OMIR33 50 and OMIR00 92. As expected, all mice contained this band.

Nucleotide sequences are listed at the bottom of the illustrations.

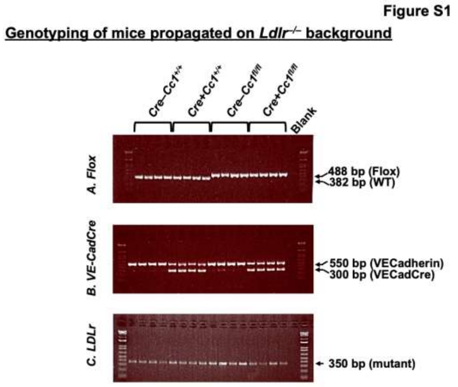

Primer sequences used for genotyping by Polymerase Chain Reaction

Gene	ID	Primer sequence (5'-3')
Flox	Flox A FP	ACACAAGGAGGCCTCTCAGATGGCG
	Flox B RP	GACTTTGGCTTCCTGACTGGAGGA
	Flox C RP	GCGCCTCCCCTACCCGGTAGAATT
Cre	VE-Cadherin Cre FP	GCAGGCAGCTCACAAAGGAACAAT
	VE-Cadherin Cre RP	ATCACTCGTTGCATCGACCGGTAA
	VE-Cadherin Gene RP	TGTCCTTGCTGAGTGACAGTGGAA
Ldlr	OMIR33 49 FP	CCATATGCATCCCCAGTCTT
	OMIR33 50 RP	GCGATGGATACACTCACTGC
	OMIR00 92 RP	AATCCATCTTGTTCAATGGCCGATC

Figure S1. Mice Genotyping. PCR amplification was performed on ear DNA using primers to detect genes for (A) Flox; (B) VE-Cadherin-Cre, (C) LDLr deletion. Nucleotide sequences are listed in the table below the agarose gels. FP denotes forward primer and RP denotes reverse primer.

Citation: Abu Helal, R.; Muturi, H.T.; Lee, A.D.; Li, W.; Ghadieh, H.E.; Najjar, S.M. Aortic Fibrosis in Insulin-Sensitive Mice with Endothelial Cell-Specific Deletion of *Ceacam1* Gene. *Int. J. Mol. Sci.* **2022**, *23*, 4335. https://doi.org/10.3390/ijms23084335

Academic Editors: Melania Manco and Amalia Gastaldelli

Received: 18 February 2022
Accepted: 10 April 2022
Published: 14 April 2022

Publisher's Note: MDPI stays neutral with regard to jurisdictional claims in published maps and institutional affiliations.

Copyright: © 2022 by the author. Licensee MDPI, Basel, Switzerland. This article is an open access article distributed under the terms and conditions of the Creative Commons Attribution (CC BY) license (https://creativecommons.org/licenses/by/4.0/).

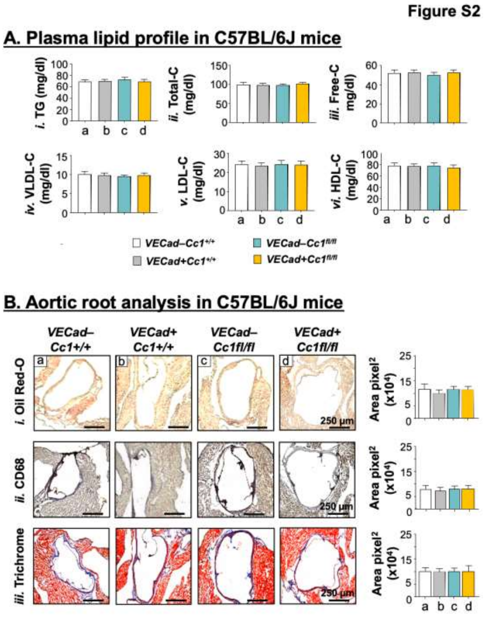

Figure S2. Lipid profiling and morphologic analysis of aortic lesions in mice on the C57BL6/J background. Male littermate mice (9 months of age, n=4/genotype) on regular chow diet were fasted overnight and their retro-orbital blood drawn. (A) plasma levels of triacylglycerol (TG) (i), and of cholesterol (–C) [total–C, Free–C, VLDL–C, LDL–C, and HDL–C (ii-vi, respectively)] were determined. (B) hearts were embedded in OCT followed by frozen sequential sectioning starting from where the aorta exits the ventricle and moving toward the aortic sinus. Aortic root lesions were analyzed by (i) Oil red-O staining (ORO), (ii) CD68 staining, and (iii) trichrome staining (n=4mice/genotype and representatives of each was included). Stained aortic areas were measured in pixel^2 using Image J, and values were expressed as mean ± SEM. Abbreviations: –C, cholesterol; –TG, triacylglycerol; OCT, optimal cutting temperature; ORO, Oil-Red O staining.

Table S1. Real-time PCR primer sequences from mouse genes

Primer	Forward Sequence (5'-3')	Reverse Sequence (5'-3')
Ang-1	CTCGTCAGACATTCATCATCCA	CACCTTCTTTAGTGCAAAGGCT
Ang-2	CAGCCACGGTCAACAACTC	CTTCTTTACGGATAGCAACCGAG
β-Catenin	TCCCTGAGACGCTAGATGAGG	CGTTTAGCAGTTTTGTCAGCTC
Cd4	TCACCTGGAAGTTCTCTGACC	GGAATCAAAACGATCAAACTGCG
Cd8	CTCTGGCTGGTCTTCAGTATGA	TCTTTGCCGTATGGTTGGTTT
Cd11b	TACGTAATTGGGGTGGGAA	GTGCCCTCAATTGCAAAGAT
Claudin-1	TGAGCCTCAGAAAAGAGCC	GCCACTAATATCGCCAGACC
Claudin-5	ATGGCGATTACGACAAGAAG	ACTGAGCAAATTCTTGCCC
Col6α3	GTCAGCTGAGTCTTGTGCTGT	ACCTAGAGAACGTTACCTCACT
Ctgf	AATGTCAGTGCGCAGCCGAAGCA	AGGGGTCACGCTCCGTACACAG
Et-1	GGTGGAAGGAAGGAAACTAC	CAAGAAGAGGCAGAAAGGCA
Etar	AACAAGTGTATGAGGACGGC	GGCCAAGATGAAGGAAAGAA
Etbr	CAGTCTTCTGCCTGGTCCTC	GGACTGCTTTTCCTCAAACG
F4/80	CAAGGAGGACAGAGTTTATCGTG	CTTTGGCTATGGGCTTCCAGTC
Fibronectin	ACGGTGTCAACTACAAGATCG	GTCTTCCCATCGTCATAGCAC
Foxp3	CCCAGGAAAGACAGCAACCTT	TTTCACAACCAGGCCACTTG
Gp91	TATGCTGATCCTGCTGCCAGT	TGTCTTCGAATCCTTGTCGAGC
Il-1β	CCCTGCAGCTGGAGAGTGTGG	TATTCTGTCCATTGAGGTGGAG
Il-6	CTTGGGACTGCCGCTGGTGA	TGCAAGTGCATCATCGTTGT
Mcp-1/Ccl2	CTTCTGGGCCTGCTGTTCA	CCAGCCTACTCATTGGGATCA
Nox4	TCCAAGCTCATTTCCCACAG	CGGAGTTCCATTACATCAGAGG
Npc-1	GGGGCATCAGTTACAATGCT	AAACACCGCACTTCCCATAG
Occludin	CTTCTGCTTCATCGCTTCC	CTTGCCCTTTCCTGCTTTC
α-Sma	CGTGGCTATTCCTTCGTTAC	TGCCAGGAGACTCCATCC
Smad7	GTTGCTGTGAATCTTACGGG	ATCTGGACAGCCTGCA
Tgfβ	GTGGAAATCAACGGGATCAG	ACTTCCAACCCAGGTCCTTC
Tlr-2	TTGCTGGGCTGACTTCTCTCA	GAAGAGTCAGGTGATGGATGTCG
Tlr-4	TCAGAACTTCAGTGGCTGGATT	AACTCTGGATAGGGTTTCCTGTCA
Tnfα	CCACCACGCTCTTCTGTCTAC	AGGGTCTGGGCCATAGAACT
Vcam-1	ATTTTCTGGGGCAGGAAGTT	ACGTGAGAACAACCGAATCC
Vegf-A	GCACATAGAGAGAATGAGCTTC	CTCCGCTCTGAACAAGGCT
Vegfr-1	TGGCTCTACGACCTTAGACTG	CAGGTTTGACTTGTCTGAGGTT
Vegfr-2	TTTGGCAAATACAACCCTTCAG	GCAGAAGATACTGTCACCACC
VE-Cadherin	CACTGCTTTGGGAGCCTTC	GGGGCAGCGATTCATTTTTCT
Zo-1	ACAAACAGCCCTACCAACC	CCATCCTCATCTTCATCTTCTTC
18S	TTCGAACGTCTGCCCTATCAA	ATGGTAGGCACGGCGACTA

Table S2. mRNA levels of inflammatory genes in the aortae of 9-month-old mice propagated on the *Ldlr*$^{-/-}$ background and fed an atherogenic high cholesterol diet in the last 3 months.

	***Ldlr*$^{-/-}$ *VECad–Cc1*$^{+/+}$**	***Ldlr*$^{-/-}$ *VECad+Cc1*$^{+/+}$**	***Ldlr*$^{-/-}$ *VECad–Cc1*$^{+/+}$**	***Ldlr*$^{-/-}$ *VECad+Cc1*$^{fl/fl}$**
Inflammation				
F4/80	0.74 ± 0.19	0.28 ± 0.07	1.20 ± 0.30	4.65 ± 1.07*†§
Cd4	0.58 ± 0.12	0.65 ± 0.11	0.64 ± 0.11	1.58 ± 0.17*†§
Cd8	1.16 ± 0.10	1.23 ± 0.13	1.07 ± 0.04	2.54 ± 0.15*†§
FoxP3	0.89 ± 0.05	0.82 ± 0.09	0.87 ± 0.04	0.89 ± 0.06
Il-1β	0.75 ± 0.06	0.72 ± 0.08	0.75 ± 0.07	1.87 ± 0.23*†§
Il-6	0.33 ± 0.09	0.53 ± 0.09	0.54 ± 0.28	1.35 ± 0.25*†§
Tnfα	0.35 ± 0.22	1.37 ± 0.18	1.32 ± 0.90	5.50 ± 0.87*†§
Mcp-1/Ccl2	1.19 ± 0.04	1.18 ± 0.13	1.58 ± 0.16	2.59 ± 0.19*†§
Tlr-2	0.29 ± 0.18	0.39 ± 0.08	0.21 ± 0.07	2.19 ± 0.69*†§
Tlr-4	0.47 ± 0.01	0.69 ± 0.12	0.52 ± 0.08	1.71 ± 0.18*†§
Cd11b	0.93 ± 0.05	1.03 ± 0.24	1.04 ± 0.07	2.31 ± 0.14*†§
Vcam-1	0.72 ± 0.27	0.75 ± 0.08	0.38 ± 0.08	2.94 ± 0.65*†§

*6-month-old mice (n≥5 per group) were fed an atherogenic HC diet for 3 months before being fasted overnight. Aortae were collected to measure mRNA content by qRT-PCR (normalized to 18S). Values were expressed as mean ± SEM. *P<0.05 vs Ldlr*$^{-/-}$*VECad–Cc1*$^{+/+,}$ †*P<0.05 vs Ldlr*$^{-/-}$*VECad+Cc1*$^{+/+}$, §*P<0.05 vs Ldlr*$^{-/-}$*VECad–Cc1*$^{fl/fl}$.

Table S3. mRNA levels of genes in the aortae of 9-month-old mice propagated on the *Ldlr*$^{-/-}$ background and fed an atherogenic diet in the last 3 months.

	VECad–Cc1$^{+/+}$ ***Ldlr***$^{-/-}$	***VECad+Cc1***$^{+/+}$ ***Ldlr***$^{-/-}$	***VECad–Cc1***$^{fl/fl}$ ***Ldlr***$^{-/-}$	***VECad+Cc1***$^{fl/j}$ ***Ldlr***$^{-/-}$
Tight junctions				
Zo-1	2.18 ± 0.40	2.37 ± 0.18	2.11 ± 0.33	0.84 ± 0.14*†§
Claudin1	2.32 ± 0.11	2.07 ± 0.15	2.01 ± 0.38	0.78 ± 0.04*†§
Claudin5	2.06 ± 0.25	2.08 ± 0.16	2.03 ± 0.37	0.69 ± 0.26*†§
Occludin	2.28 ± 0.05	2.89 ± 0.32	2.45 ± 0.08	0.61 ± 0.06*†§
Vascular integrity				
VE-Cadherin	1.31 ± 0.12	1.23 ± 0.18	1.59 ± 0.39	0.50 ± 0.05*†§
β-Catenin	1.05 ± 0.04	1.29 ± 0.26	1.09 ± 0.12	0.48 ± 0.07*†§
Vegfr-1	2.70 ± 0.57	2.54 ± 0.42	2.31 ± 0.09	0.84 ± 0.23*†§
Vegfr-2	1.60 ± 0.29	1.84 ± 0.14	1.29 ± 0.067	0.30 ± 0.14*†§
Vegf-A	3.26 ± 0.43	3.65 ± 0.41	3.29 ± 0.28	1.57 ± 0.28*†§
Ang-1	2.33 ± 0.49	2.11 ± 0.11	2.67 ± 0.41	0.86 ± 0.04*†§
Ang-2	1.36 ± 0.17	1.49 ± 0.19	1.55 ± 0.31	0.27 ± 0.16*†§
Oxidative stress				
Nox4	2.40 ± 0.05	2.98 ± 0.27	2.34 ± 0.42	4.52 ± 0.07*†§
Gp91	1.32 ± 0.12	1.467 ± 0.38	2.37 ± 0.51	4.16 ± 0.49*†§
Npc-1	1.08 ± 0.09	1.09 ± 0.11	1.05 ± 0.12	0.62 ±0.07*†§
Fibrosis				
Fibronectin	1.44 ± 0.24	1.55 ± 0.29	0.98 ± 0.16	5.14 ± 0.29*†§
Ctgf	1.41 ± 0.15	1.41 ± 0.08	1.14 ± 0.26	5.53 ± 1.20*†§
α-Sma	1.94 ± 0.32	2.13 ± 0.30	1.34 ± 0.33	7.25 ± 1.21*†§
Col6-α3	1.29 ± 0.11	1.29 ± 0.13	1.53 ± 0.27	3.14 ± 0.06*†§
Tgf-β	0.80 ± 0.08	0.48 ± 0.41	0.94 ± 0.10	5.04 ± 0.29*†§
Smad7	1.49 ± 0.14	1.58 ± 0.23	1.82 ± 0.39	0.54 ± 0.01*†§
Et-1	1.75 ± 0.65	1.04 ± 0.20	1.30 ± 0.13	3.30 ± 0.29*†§
Etar	1.05 ± 0.20	0.89 ± 0.40	1.04 ± 0.49	3.44 ± 0.41*†§
Etbr	2.36 ± 0.51	2.19 ± 0.17	2.14 ± 0.23	0.76 ± 0.10*†§
Etar/Etbr	0.64 ± 0.20	0.22 ± 0.07	0.60 ± 0.27	3.10 ± 0.85*†§

*6-month-old male mice (n≥5 per group) were fed an atherogenic diet for 3 months before being fasted overnight. Aortae were collected to measure mRNA content by qRT-PCR (normalized to 18S). Values were expressed as mean ± SEM. *P<0.05 vs Ldlr*$^{-/-}$*VECad–Cc1*$^{+/+,}$ †*P<0.05 vs Ldlr*$^{-/-}$*VECad+Cc1*$^{+/+}$, §*P<0.05 vs Ldlr*$^{-/-}$*VECad–Cc1*$^{fl/fl}$.
